# Efficacy and safety of Lanqin Oral Liquid combined with Western medicine for the treatment of pharyngitis: a systematic review and meta-analysis

**DOI:** 10.3389/fmed.2026.1820924

**Published:** 2026-05-13

**Authors:** Xiangdong Mu, Qiongzhen Luo, Yasheng Yuan

**Affiliations:** 1 Department of Respiratory and Critical Care Medicine, Beijing Tsinghua Changgung Hospital, Tsinghua University, Beijing, China; 2 Department of Respiratory and Critical Care Medicine, Beijing Tsinghua Changgung Hospital, School of Clinical Medicine, Tsinghua University, Beijing, China; 3 Department of Otorhinolaryngology Head and Neck Surgery, Huashan Hospital Fudan University, Shanghai, China

**Keywords:** efficacy, Lanqin Oral Liquid, pharyngitis, safety, Western medicine

## Abstract

**Background:**

This study aimed to systematically review and meta-analyze the efficacy and safety of Lanqin Oral Liquid combined with Western medicine for the treatment of pharyngitis.

**Methods:**

A comprehensive and systematic literature search was conducted by two independent reviewers across six electronic databases–PubMed, Embase, Web of Science, CNKI, WanFang and VIP. Outcomes included the effective rate, symptoms improvement, C-reactive protein (CRP) levels, and the incidence of adverse events. The Cochrane Risk of Bias tool was adopted to evaluate the quality of the included studies, and RevMan 5.4 and Stata 15.0 software were used for meta-analysis.

**Results:**

A total of 12 studies were included. The effective rate of Lanqin Oral Liquid combined with Western medicine for pharyngitis was significantly higher than that of Western medicine monotherapy (OR = 3.68, 95% CI [2.55, 5.33], *Z* = 6.92, *P* < 0.001), with no heterogeneity across studies (I^2^ = 0%, *P* = 0.73). Compared with Western medicine monotherapy, combination therapy significantly shortened the disappearance time of pharyngeal redness/swelling, sore throat, and phlegm in patients with acute pharyngitis, and reduced Reflux Symptom Index score and Reflux Finding Score in those with chronic pharyngitis (all *P* < 0.05). The combination therapy was significantly superior to Western medicine monotherapy in reducing CRP levels (SMD = −2.72, 95% CI [−3.37, −2.07], *Z* = 8.19, *P* < 0.001). The incidence of adverse events showed no significant difference between the two groups (OR = 0.63, 95% CI [0.28, 1.45], *Z* = 1.08, *P* = 0.28).

**Conclusion:**

For pharyngitis patients, the combination of Lanqin Oral Liquid and Western medicine may provide better efficacy compared with Western medicine monotherapy, and its safety profile should be interpreted with caution owing to limited available evidence.

## 1 Introduction

Pharyngitis is a common upper-respiratory-tract disorder that affects the mucosa of the oropharynx and adjacent tissues. Its high incidence across all age groups makes it a major clinical concern (1, 2). The condition is broadly classified as acute or chronic, each form having distinct features and etiologies. Acute pharyngitis is typically triggered by viral or bacterial infections, or physical/chemical irritants. It is primarily characterized by sore throat, erythema, swelling, and dysphagia (3), and is generally self-limiting, resolving spontaneously within 1 week (4, 5). Chronic pharyngitis, in contrast, is a persistent inflammatory state lasting ≥3 months or recurring intermittently (6). It presents with dry throat, dry cough or pharyngeal foreign body sensation, itching pain and discomfort, scanty phlegm (7). These symptoms often arise from long-standing inflammation, heightened neural sensitivity, or altered mucus secretion, and may be aggravated by environmental irritants or comorbidities such as gastroesophageal reflux disease (GERD) (8, 9).

Currently, the treatment strategies for pharyngitis primarily rely on Western medicines (antibiotics, antivirals, and glucocorticoids), yet their inherent limitations restrict clinical utility and long-term efficacy (10). Antibiotics are effective only against bacterial pharyngitis and are ineffective for the viral cases that account for 80% of acute cases (11, 12). Misuse of antibiotics accelerates multidrug resistance and can cause adverse events and dysbiosis (13, 14). Antivirals possess a narrow spectrum and a limited therapeutic window (48–72 h), and may cause hepatotoxicity or nephrotoxicity (15, 16). Glucocorticoids afford rapidly relieve severe symptoms such as pharyngeal edema, yet they neither eradicate the pathogen nor shorten illness duration and may suppress local immunity, masking progressive infection and inviting secondary complications (17). These shortcomings underscore the urgent need for safer, mechanism-based alternatives–either from traditional Chinese medicine (TCM) or integrated TCM-Western medicine regimens.

Traditional Chinese medicine classifies pharyngitis as “throat obstruction” and advocates multi-target modulation through compound herbal formulas that clear heat, detoxify, open the lungs, and dispel phlegm (18). Lanqin Oral Liquid, a classic TCM-based preparation, has demonstrated efficacy in the management of pharyngitis through its anti-inflammatory, antiviral, and detoxifying effects. Its formulation primarily consists of five natural: *Isatis tinctoria* L., *Phellodendron chinense* C.K.Schneid., *Gardenia jasminoides* J.Ellis, *Sterculia lychnophora* Hance, and *Scutellaria baicalensis* Georgi (19, 20). These components exert synergistic effects to relieve pharyngitis symptoms: *Gardenia jasminoides* J.Ellis clears heat and reduces swelling to relieve sore throat; *Scutellaria baicalensis* Georgi and *Phellodendron chinense* C.K.?Schneid. eliminate toxins and alleviate inflammation by clearing heat and drying dampness; and *Isatis tinctoria* L. exerts antiviral and antibacterial effects, further mitigating pharyngeal swelling (21). Currently, Lanqin Oral Liquid is widely used in treating pharyngitis, demonstrating remarkable clinical efficacy with no obvious toxicity or emergence of drug resistance (22).

Furthermore, preliminary reports have hinted that adding Lanqin Oral Liquid to standard Western therapy achieves better clinical outcomes and comparable or superior safety in both acute and chronic pharyngitis (23–26). However, these findings remain fragmented across individual studies with limited sample sizes, and a definitive, high-level evidence-based synthesis is lacking. Therefore, this systematic review and meta-analysis was conducted to determine, with robust evidence, whether the combination of Lanqin Oral Liquid with Western medicine surpasses Western monotherapy in efficacy and safety for pharyngitis.

## 2 Methods

This systematic review and meta-analysis was conducted in accordance with the Preferred Reporting Items for Systematic Reviews and Meta-Analyses (PRISMA) guidelines (27).

### 2.1 Search strategy

A comprehensive and systematic literature search was conducted by two independent reviewers across six electronic databases–PubMed, Embase, Web of Science, China National Knowledge Infrastructure (CNKI), WanFang and VIP. The search covered the period from database inception to 30 September, 2025 and was restricted to publications in English or Chinese. The search terms included “Lanqin Oral Liquid,” “pharyngitis,” “chronic pharyngitis” and “acute pharyngitis.”

### 2.2 Inclusion and exclusion criteria

The inclusion criteria were as follows: (1) patients diagnosed with either acute or chronic pharyngitis; (2) the experimental group received Lanqin Oral Liquid combined with Western medicine, whereas the control group received Western medicine monotherapy; (3) the outcomes included effective rate, symptoms improvement, inflammatory marker C-reactive protein (CRP) and adverse events; (4) randomized controlled trials (RCTs) or clinical controlled studies. The exclusion criteria were as follows: (1) duplicate publications; (2) conference abstracts, reviews, letters, and existing systematic reviews or meta-analyses; (3) studies with incomplete or unusable outcome data; (4) animal studies.

### 2.3 Data extraction and risk of bias assessment

Two reviewers (X.M. and Q.L.) independently screened all studies by title and abstract, with potentially relevant studies then subjected to full-text review. For each eligible study, data were extracted with a pre-designed form capturing: first author, publication date, sample size; patients’ age; disease type; treatment regimen (drug, dose, and treatment duration); and outcomes. The quality of the included studies was assessed by using a Cochrane Risk of Bias tool. Six domains were evaluated: selection bias, performance bias, detection bias, attrition bias, reporting bias, and other potential sources of bias. Each domain was rated as “low risk,” “high risk,” or “unclear risk.” Any disagreements at either stage were resolved through discussion, and a third reviewer (Y.Y.) was consulted when necessary.

### 2.4 Statistical analysis

All analyses were performed using RevMan 5.4 and Stata 15.0 software. Dichotomous variables were expressed as the odds ratios (ORs) with 95% confidence intervals (CIs), whereas continuous variables were expressed as the standardized mean differences (SMDs) with 95% CIs. The Q-test and I^2^ statistic were utilized to assess the heterogeneity across the included studies. A fixed-effect model was used when *P* > 0.1 and I^2^ ≤ 50%. Otherwise, a random-effects model was adopted. Funnel plots with Egger test were employed to assess publication bias. Sensitivity analysis was conducted to assess the robustness of the results. A two-sided *P* < 0.05 was considered statistically significant.

## 3 Results

### 3.1 Study selection

**FIGURE 1:**
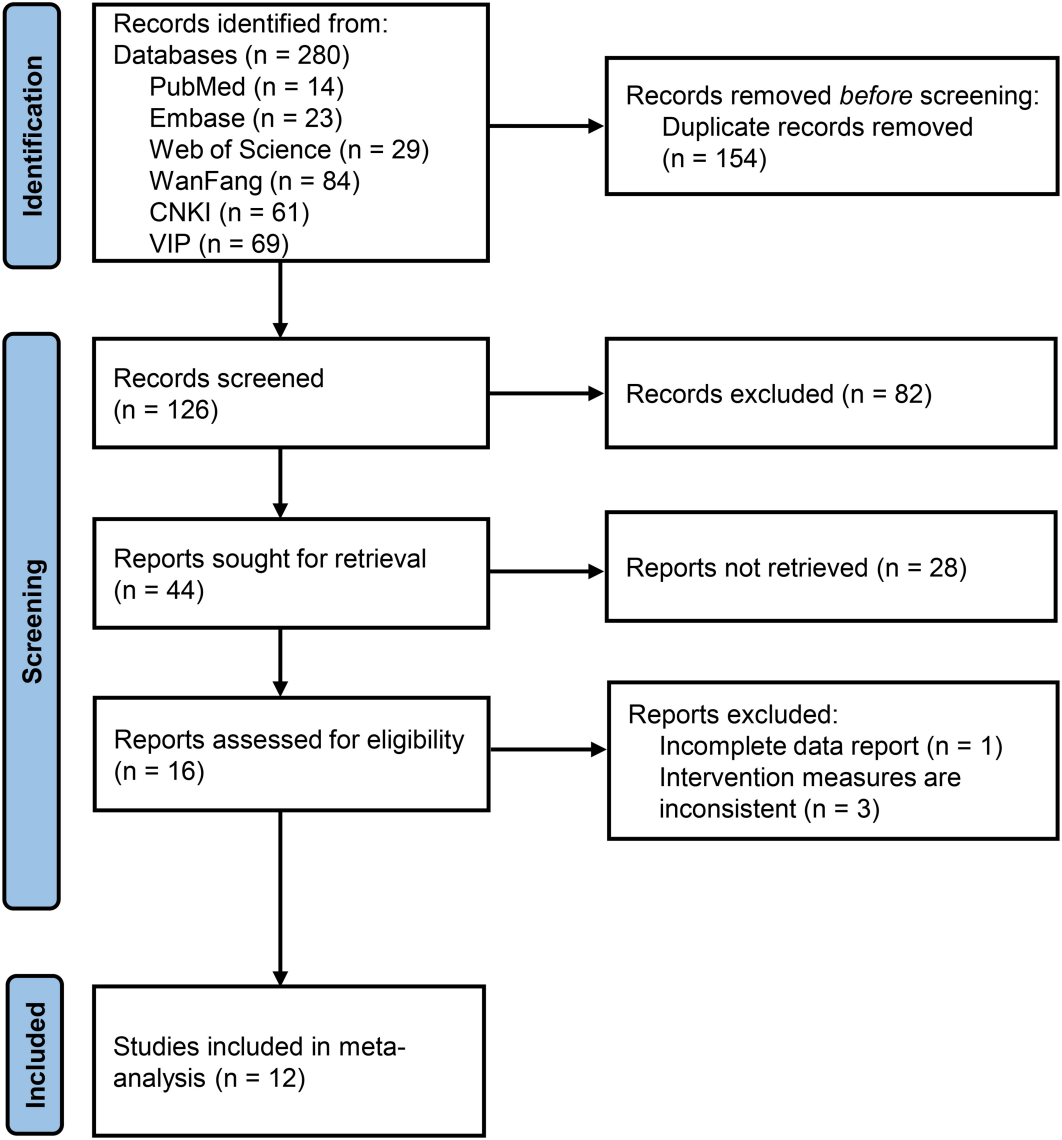
Preferred Reporting Items for Systematic Reviews and Meta-Analyses (PRISMA) flow diagram of study selection.

As shown in Figure 1, a total of 280 records were identified across six electronic databases: PubMed (*n* = 14); Embase (*n* = 23); Web of Science (*n* = 29); WanFang (*n* = 84); CNKI (*n* = 61); and VIP (*n* = 69). After duplicate removal (*n* = 154), 126 studies remained for the title and abstract selection. After screening, 82 irrelevant studies were excluded, and 44 studies were selected for full-text assessment–of which 28 full-texts were unretrievable. Subsequently, 16 full-text studies were further assessed for eligibility, among which 4 were excluded for specific reasons. Ultimately, 12 studies were considered eligible for inclusion.

### 3.2 Study characteristics and quality assessment

The characteristics of the included studies are presented in Table 1. Among the 12 studies, 4 studies focused on acute pharyngitis, while 8 studies addressed chronic pharyngitis. The total sample included 1362 patients, with sample sizes ranging from 50 to 500 patients. Regarding the intervention regimens, the specific Western medicines used across the studies were as follows: cefixime granules (3 studies) (23, 28, 29), amoxicillin and clavulanate potassium dispersible tablets (1 study) (26), esomeprazole magnesium (2 studies) (24, 30), and rabeprazole sodium enteric-coated capsules (6 studies) (25, 31–35).

**TABLE 1:** Basics characteristics of included studies.

Author (year)	Sample size	Age (years)	Disease type	Intervention	Outcomes
Jiang et al. (23)					
Experimental group	31	6.51 ± 1.11	Acute pharyngitis	Lanqin Oral Liquid, 10 ml/dose, bid; +Western medicine; 7 d	a, b, c, d
Control group	31	6.25 ± 1.03		Cefixime granules, 2 mg/kg, bid; 7 d	
Fu et al. (28)					
Experimental group	46	6.11 ± 0.23	Acute pharyngitis	Lanqin Oral Liquid, 20 ml/dose, tid; +Western medicine; 7 d	a, b, c, d
Control group	46	4.24 ± 0.36		Cefixime granules, 1.5–3 mg/kg, bid; 7 d	
Zhu et al. (29)					
Experimental group	250	32.58 ± 6.90	Acute pharyngitis	Lanqin Oral Liquid, 20 ml/dose, tid; +Western medicine; 7 d	a, b, c, d, f
Control group	250	32.95 ± 6.68		Cefixime granules, 1.5–3 mg/kg, bid; 7 d	
Hua et al. (26)					
Experimental group	43	40.5 ± 3.6	Acute pharyngitis	Lanqin Oral Liquid, 20 ml/dose, tid; +Western medicine; 5 d	a
Control group	43	40.8 ± 3.4		Amoxicillin and clavulanate potassium dispersible tablets, 457 mg/dose, tid; 5 d	
Cheng et al. (30)					
Experimental group	40	22–58	Chronic pharyngitis	Lanqin Oral Liquid, 20 ml/dose, tid; +Western medicine; 4 w	a
Control group	40	22–58		Esomeprazole magnesium, 20 mg/dose, qd; 4 w	
Fan et al. (24)					
Experimental group	40	37–80	Chronic pharyngitis	Lanqin Oral Liquid, 20 ml/dose, tid; +Western medicine; 4 w	a
Control group	40	37–80		Esomeprazole Magnesium, 20 mg/dose, qd; 4 w	
Yu et al. (25)					
Experimental group	33	35.62 ± 7.51	Chronic pharyngitis	Lanqin Oral Liquid, 20 ml/dose, tid; +Western medicine; 4 w	a
Control group	33	37.12 ± 6.49		Rabeprazole sodium enteric coated capsules, 10 mg/dose, qd; 4 w	
Wang et al. (31)					
Experimental group	60	63.4 ± 5.1	Chronic pharyngitis	Lanqin Oral Liquid, 20 ml/dose, tid; +Western medicine; 1 w	a, b, e, f
Control group	60	63.2 ± 5.9		Rabeprazole sodium enteric coated capsules, 20 mg/dose, bid; 1 w	
Liu et al. (32)					
Experimental group	30	46.18 ± 3.29	Chronic pharyngitis	Lanqin Oral Liquid, 20 ml/dose, tid; +Western medicine; 4 w	a, b, e, f
Control group	30	46.97 ± 3.08		Rabeprazole, 10 mg/dose, qd; 4 w	
Fan et al. (33)					
Experimental group	36	36.73 ± 7.62	Chronic pharyngitis	Lanqin Oral Liquid, 20 ml/dose, tid; +Western medicine; 4 w	a
Control group	36	38.23 ± 7.51		Rabeprazole sodium enteric coated capsules, 10 mg/dose, qd; 4 w	
Fu et al. (34)					
Experimental group	47	45.4 ± 4.3	Chronic pharyngitis	Lanqin Oral Liquid, 20 ml/dose, tid; +Western medicine; 4 w	a
Control group	47	45.6 ± 4.6		Rabeprazole sodium enteric coated capsules, 10 mg/dose, qd; 4 w	
Bian et al. (35)					
Experimental group	25	45.50 ± 19.50	Chronic pharyngitis	Lanqin Oral Liquid, 20 ml/dose, tid; +Western medicine; 4 w	a
Control group	25	46.76 ± 19.24		Rabeprazole sodium enteric coated capsules, 10 mg/dose, qd; 4 w	

a, effective rate; b, disappearance time of clinical symptoms (pharyngeal erythema and swelling, sore throat, and cough); c, CRP; d, adverse events; e, Reflux Symptom Index (RSI) RIS; f, Reflux Finding Score (RFS). qd, once a day; bid, twice a day; tid, three times a day.

**FIGURE 2:**
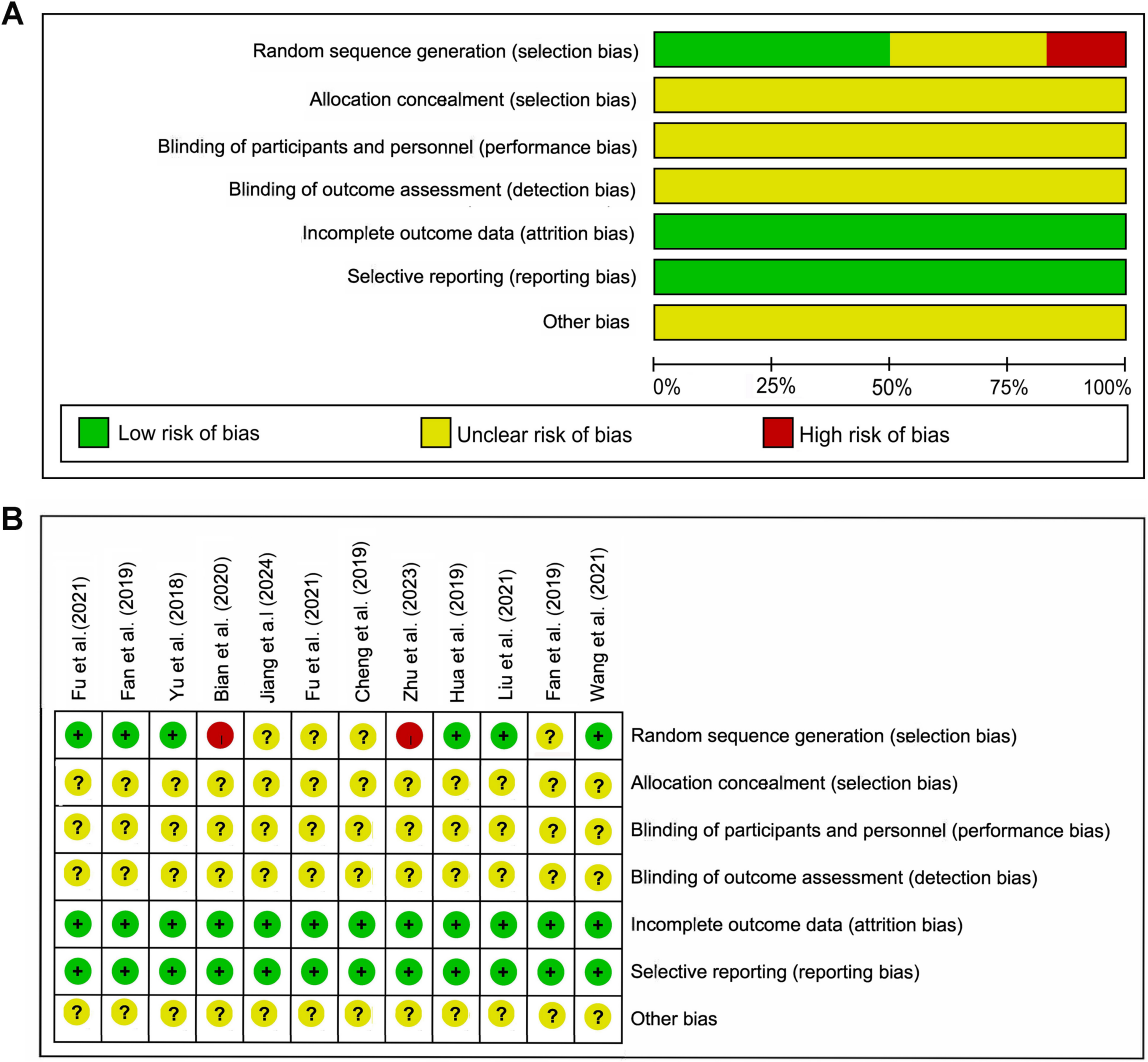
Quality assessment. **(A)** Risk of bias graph. **(B)** Risk of bias summary.

The results of the risk of bias assessment for the included studies are presented in Figure 2. Regarding the randomization process, six studies were rated as low risk, two studies were rated as high risk, and four studies were judged as having an unclear risk. None of the studies reported the method of concealment of random allocation and blinding. All studies consistently showed a low risk of bias in the domains related to deviations from incomplete outcome data and selective reporting. Additionally, the risk of bias for other potential sources of bias was unclear.

### 3.3 Primary outcomes

**FIGURE 3:**
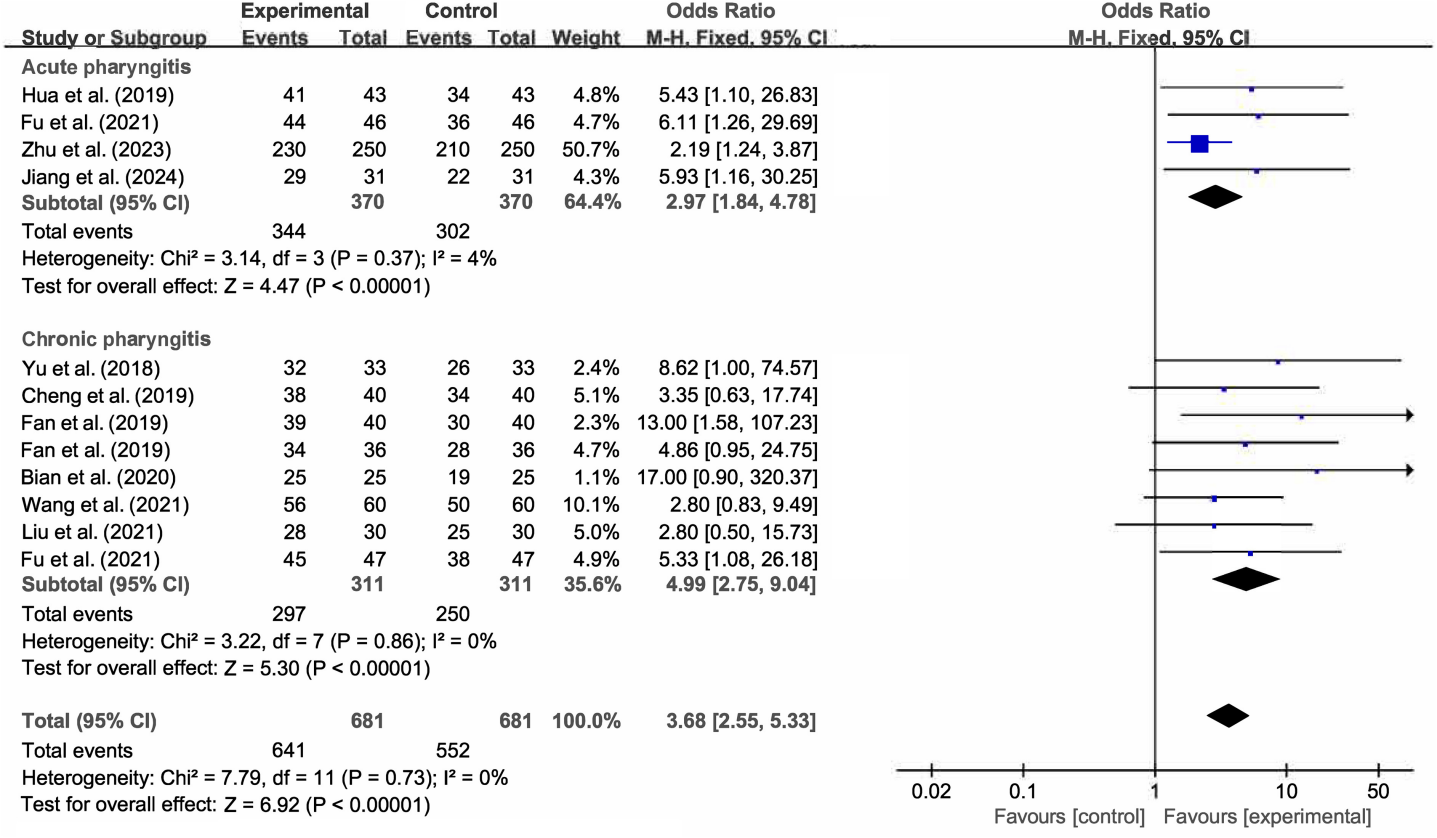
Forest plots of meta-analysis for the effective rate of combination therapy *vs.* Western medicine.

**FIGURE 4:**
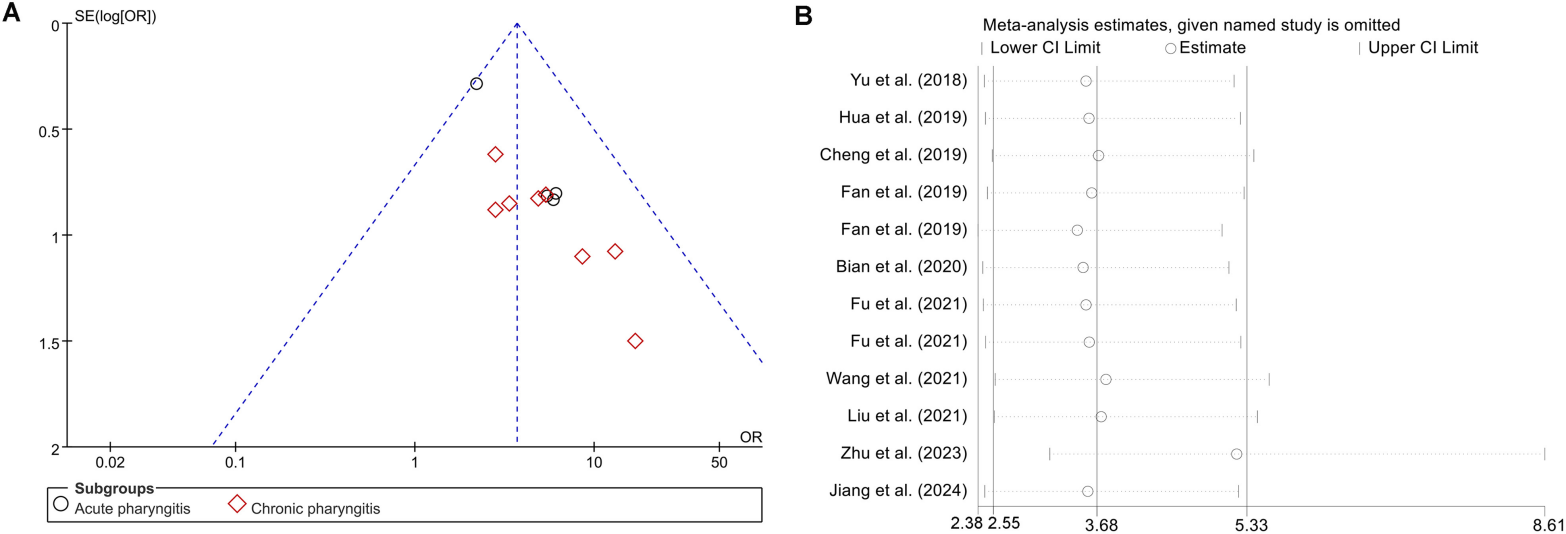
**(A)** Analysis of publication bias with the funnel plot about the effective rate. **(B)** Sensitivity analysis of effective rate.

The primary outcomes focused on the effective rate. All 12 studies reported the effective rate of Lanqin Oral Liquid combined with Western medicine versus Western medicine monotherapy in the treatment of pharyngitis. The fixed-effect model meta-analysis revealed that the effective rate of the combination therapy for pharyngitis was significantly higher than that of Western medicine monotherapy (OR = 3.68, 95% CI [2.55, 5.33], *Z* = 6.92, *P* < 0.001). There was no heterogeneity among studies (I^2^ = 0%, *P* = 0.73, Figure 3). Meanwhile, the funnel plot showed an approximately symmetrical distribution, suggesting a low risk of publication bias or small-study effects (Figure 4A). Furthermore, as shown in Figure 4B, we verified the stability of the results by excluding one study at a time and found that there was no significant change in the overall effect magnitude of each group.

To further investigate the results, the subgroup analysis was performed based on disease type. In acute pharyngitis subgroup, the combination therapy significantly improved the effective rate compared to Western medicine monotherapy (OR = 2.97, 95% CI [1.84, 4.78], *Z* = 4.47, *P* < 0.001). Similarly, in the chronic pharyngitis subgroup, the combination therapy also exerted a statistically significant therapeutic advantage (OR = 4.99, 95% CI [2.75, 9.04], *Z* = 5.30, *P* < 0.001). No significant heterogeneity was observed across studies in either subgroup analysis (I^2^ = 4%, *P* = 0.37; I^2^ = 0%, *P* = 0.86, respectively; Figure 3). These findings confirmed that the therapeutic benefit of combination therapy was consistent across both acute and chronic pharyngitis subgroups.

### 3.4 Secondary outcomes

#### 3.4.1 Improvement of clinical symptoms

**FIGURE 5:**
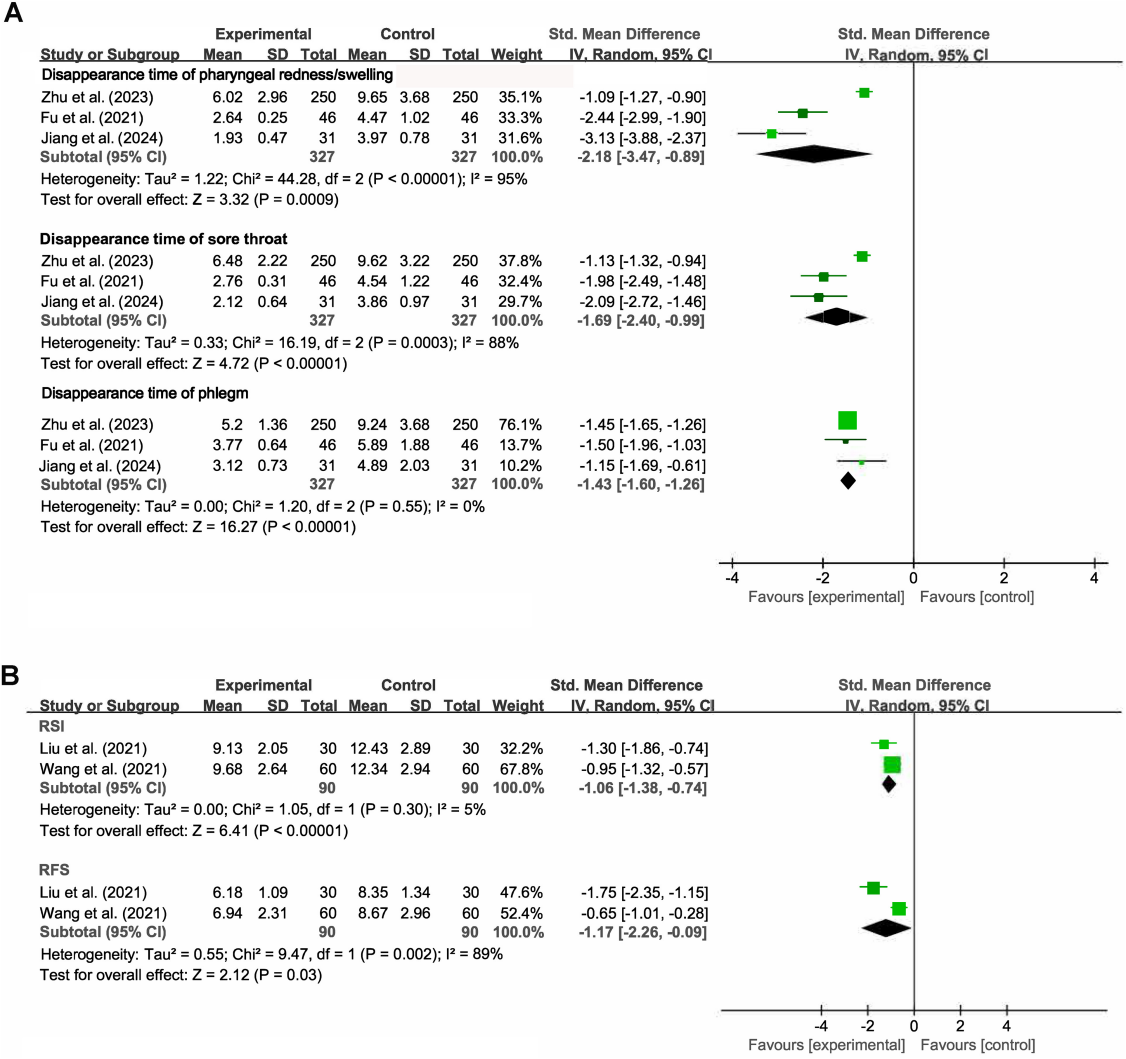
Forest plots of meta-analysis for symptom improvement in panel **(A)** the acute pharyngitis subgroup and **(B)** the chronic pharyngitis subgroup.

Meta-analysis for symptoms improvement was performed separately for acute and chronic pharyngitis. The random-effects model was utilized due to the small number of studies introducing potential heterogeneity. For acute pharyngitis (3 studies), the meta-analysis showed that the combination therapy significantly shortened the disappearance time of several key symptoms compared to Western medicine monotherapy: pharyngeal redness and swelling (SMD = −2.18, 95% CI [−3.47, −0.89], *Z* = 3.32, *P* < 0.001; I^2^ = 95%, *P* < 0.001), sore throat (SMD = −1.69, 95% CI [−2.40, −0.99], *Z* = 4.72, *P* < 0.001; I^2^ = 88%, *P* < 0.001) and phlegm in the throat (SMD = −1.43, 95% CI [−1.60, −1.26], *Z* = 16.27, *P* < 0.001; I^2^ = 0%, *P* = 0.55; Figure 5A). For chronic pharyngitis (2 studies), the meta-analysis also confirmed substantial therapeutic efficacy of the combination therapy. Specifically, compared with Western medicine monotherapy, the combination therapy yielded significantly reduced scores on both the Reflux Symptom Index (RSI; SMD = −1.06, 95% CI [−1.38, −0.74], *Z* = 6.41, *P* < 0.001; I^2^ = 5%, *P* = 0.30) and the Reflux Finding Score (RFS; SMD = −1.17, 95% CI [−2.26, −0.09], *Z* = 2.12, *P* = 0.03; I^2^ = 89%, *P* = 0.002; Figure 5B). Given the extremely high between-study heterogeneity across several symptom outcomes, these results should be interpreted with caution, which may be attributed to differences in patients’ characteristics, treatment regimens, and the standards of outcome assessment. Sensitivity analysis was not feasible due to the small number of included studies.

#### 3.4.2 Inflammatory marker CRP

**FIGURE 6:**
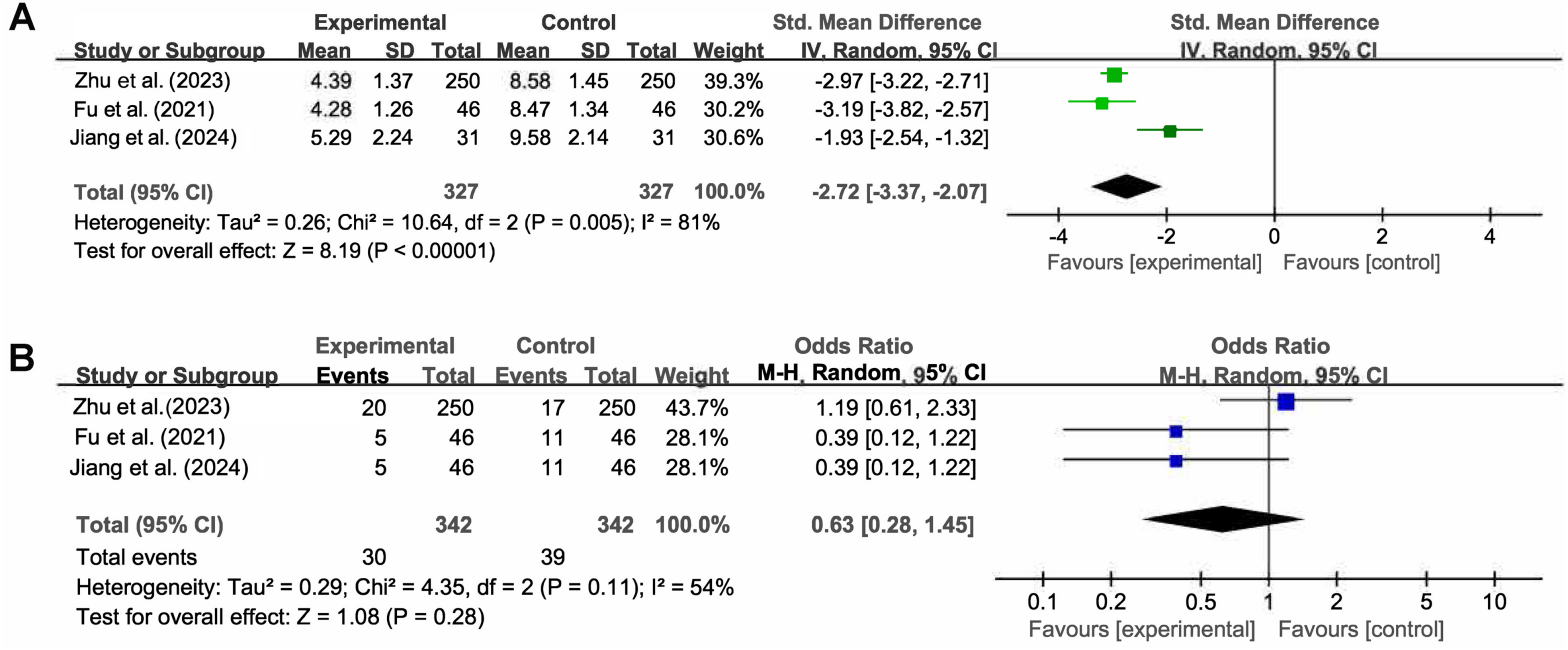
Forest plots of meta-analysis for panel **(A)** the level of C-reactive protein and **(B)** the incidence of adverse events.

Three studies reported data on inflammatory marker CRP. High heterogeneity was detected (I^2^ = 81%, *P* = 0.005), which may stem from variations in baseline inflammation levels and concomitant medications. A random-effects model was therefore used. The pooled results revealed that the combination therapy exhibited a statistically significant superiority over Western medicine monotherapy in reducing CRP levels (SMD = −2.72, 95% CI [−3.37, −2.07], *Z* = 8.19, *P* < 0.001; Figure 6A).

#### 3.4.3 Incidence of adverse events

Three studies reported the incidence of adverse events. The most commonly reported adverse events were gastrointestinal symptoms (including diarrhea, nausea, and gastric discomfort) and skin rash, which were consistent across the included studies. Moderate heterogeneity was observed across studies (I^2^ = 54%, *P* = 0.11), warranting the use of a random-effects model. The meta-analysis demonstrated no statistically significant difference in the overall incidence of adverse events between the combination therapy and Western medicine monotherapy (OR = 0.63, 95% CI [0.28, 1.45], *Z* = 1.08, *P* = 0.28; Figure 6B). Given the small number of included studies and limited sample size, this analysis was insufficiently powered to detect rare adverse events or potential herb-drug interactions, and thus the safety findings should be interpreted with caution.

## 4 Discussion

This systematic review and meta-analysis confirmed that adding Lanqin Oral Liquid to Western medicine confers a clinically significant advantage over Western medicine monotherapy for pharyngitis. These findings underscored the therapeutic synergy achievable through integrating Western medicine and multi-component TCM. Western medicine targets specific etiologies (36–38), whereas Lanqin Oral Liquid delivers broad-spectrum anti-inflammatory, antiviral, and immunomodulatory effects, providing a more comprehensive therapeutic response (20). The convergence of these distinct mechanisms offers a compelling strategy to overcome the limitations of conventional monotherapy–most notably, antibiotic resistance. By synthesizing the available evidence, this meta-analysis furnished a higher level of validation for the combination therapy, which might present a valuable advancement in the evidence-based management of pharyngitis.

Cefixime, an oral third-generation cephalosporin, is indicated for adults and pediatric patients (≥6 months) with acute pharyngitis caused by susceptible isolates of *Streptococcus pyogenes* (36, 39). The PPIs, such as rabeprazole and esomeprazole, are the first-line agents for GERD-related chronic pharyngitis (37, 38, 40). Amoxicillin/clavulanate potassium, a broad-spectrum antibiotic, is widely recognized as an effective treatment for streptococcal pharyngitis (41). Notably, these four Western medicines were collectively incorporated into the therapeutic regimens of the studies included in this meta-analysis. The pooled results demonstrated that the combination therapy significantly outperformed Western medicine monotherapy in terms of effective rate. This superiority may be attributed to the synergistic capacity of the combination therapy to target both the etiology (e.g., infection, reflux) and inflammatory manifestations of pharyngitis. Furthermore, subgroup analyses consistently demonstrated this beneficial effect in both acute and chronic pharyngitis. The findings offer robust evidence to support the combined use of Lanqin Oral Liquid with appropriate Western medicine tailored to the specific subtype of pharyngitis.

Pharyngitis-related symptoms exert a substantial impact on patients’ well-being and quality of life, so effective symptom control is a key clinical goal (10). This meta-analysis demonstrated that combination therapy delivers multidimensional benefits in this regard. For acute pharyngitis patients, it significantly accelerated the relief of core symptoms, such as pharyngeal redness and swelling, sore throat and phlegm in the throat, enabling a quicker return to normal activities. In the management of chronic pharyngitis, the combination therapy produced clinically meaningful reductions in both the RSI and RFS. This is particularly significant, as these improvements target the often-overlooked laryngopharyngeal reflux component–an underlying pathogenic mechanism in many treatment-resistant cases (40, 42). By tackling this comorbidity, combination therapy may be beyond mere symptomatic control toward modifying the disease process.

Importantly, TCM formulations possess unique complementary advantages in inhibiting inflammatory cascades owing to multi-component synergistic effects (13). In the present meta-analysis, combination therapy significantly inhibited systemic inflammation, as evidenced by reduced CRP levels compared with Western medicine monotherapy. As a well-recognized serum marker of systemic inflammation, CRP is closely linked to the severity of pharyngeal mucosal injury and inflammatory responses in pharyngitis. This reduction in CRP levels was paralleled by improved clinical outcomes, including faster remission of sore throat, pharyngeal swelling, and other core symptoms, as well as enhanced overall therapeutic efficacy. These clinical findings are consistent with the anti-inflammatory properties of Lanqin Oral Liquid that have been reported in previous non-clinical and clinical studies (21). Collectively, these results suggest that combination therapy is not merely a strategy for rapid symptom control but also a comprehensive approach that targets both the manifestations and mechanisms of pharyngitis.

Safety analysis revealed that the combination of Lanqin Oral Liquid and cefixime appeared to be generally well tolerated in the included studies. Cefixime, as a β-lactam antibiotic, is known to be associated with common adverse events, such as gastrointestinal discomfort and allergic responses (39). Notably, no obvious exacerbation of such adverse events was observed when cefixime was co-administered with Lanqin Oral Liquid. Moreover, no herb-drug interactions or cephalosporin-related anaphylaxis were reported in the included studies. However, these safety findings should be interpreted with caution, as only three studies reported adverse events and moderate between-study heterogeneity was observed. The observed variability may be attributable to differences in infection severity, dosage regimens of cefixime and Lanqin Oral Liquid, treatment duration, as well as patient characteristics and comorbidity burden across studies.

Several limitations of the meta-analysis should be acknowledged. First, although six databases in Chinese and English were systematically searched, large sample and multicenter studies were still lacking. Second, all included studies were conducted in China, potentially limiting the generalizability of the findings to other populations and healthcare contexts. Third, most included studies had unclear or inadequate allocation concealment and blinding methods, which may increase the risk of bias and affect the confidence in the findings. Fourth, some outcomes were derived from only 2 to 4 studies with substantial heterogeneity observed, potentially compromising the precision and robustness of effect size estimates. Fifth, the relatively short treatment durations (1–4 weeks) in the included studies precluded conclusions regarding long-term effectiveness and safety.

## 5 Conclusion

This systematic review and meta-analysis demonstrates that the combination of Lanqin Oral Liquid and Western medicine may provide better efficacy to Western medicine monotherapy for the treatment of pharyngitis patients, and its safety profile should be interpreted with caution owing to limited available evidence. Large-scale, high-quality RCTs are needed to further validate these findings.

## Data Availability

The original contributions presented in this study are included in this article/supplementary material, further inquiries can be directed to the corresponding author.
